# Synima: a Synteny imaging tool for annotated genome assemblies

**DOI:** 10.1186/s12859-017-1939-7

**Published:** 2017-11-21

**Authors:** Rhys A. Farrer

**Affiliations:** 1 Department of Infectious Disease Epidemiology, Imperial College London, London, W2 1PG UK; 2 Department of Genetics, Environment and Evolution, University College London, London, WC1E 6BT UK

**Keywords:** Synteny, Imaging tool, Orthology, Visualization

## Abstract

**Background:**

Ortholog prediction and synteny visualization across whole genomes are valuable methods for detecting and representing a range of evolutionary processes such as genome expansion, chromosomal rearrangement, and chromosomal translocation. Few standalone methods are currently available to visualize synteny across any number of annotated genomes.

**Results:**

Here, I present a Synteny Imaging tool (Synima) written in Perl, which uses the graphical features of R. Synima takes orthologues computed from reciprocal best BLAST hits or OrthoMCL, and DAGchainer, and outputs an overview of genome-wide synteny in PDF. Each of these programs are included with the Synima package, and a pipeline for their use. Synima has a range of graphical parameters including size, colours, order, and labels, which are specified in a config file generated by the first run of Synima – and can be subsequently edited. Synima runs quickly on a command line to generate informative and publication quality figures. Synima is open source and freely available from https://github.com/rhysf/Synima under the MIT License.

**Conclusions:**

Synima should be a valuable tool for visualizing synteny between two or more annotated genome assemblies.

## Background

Orthologous genes are sections of nucleic acid that encode a protein or functional RNA molecule and have descended from a single ancestral gene followed by divergence through speciation [1, 2]. In contrast, paralogous genes are those that have arisen from duplication within a single species. Orthology and paralogy together constitute sequence homology. Numerous repositories of pre-determined orthologs are available including OrthoDB [3], Eggnog [4], InParanoid [5], and the Orthologous Matrix (OMA) project [6]. Orthologous genes can also be identified de novo from newly annotated genomes to assess assembly or annotation completeness, predict/infer gene function, and as a precursor to phylogenetic analyses between two or more species [7–9]. Many tools and methods have been developed to predict orthologs, for example via reciprocal best hits from pairwise Basic Local Alignment Search Tool (BLAST) [10] of proteins, which can be further clustered and assessed by such tools (as well as both being databases): InParanoid [11] or OrthoMCL [12]. Large gene families, low quality annotation and/or assemblies have each been identified as contributing factors to accuracy in ortholog prediction [13]. Ortholog predictions are further refined by identifying those that fall in contiguous chains, such as by the tool DAGchainer [14].

Orthologs can be used to provide evidence for synteny: the conservation of the ordering of loci on chromosomes between two individuals or species. Visualizing syntenic regions is valuable for detecting and displaying evolutionary processes, including genome expansions [15], and chromosomal translocations [16]. Furthermore, lack of synteny has been used to identify horizontal gene transfer [17]. Genome assembly contamination or inaccuracies may also be detected given, for example, low levels of synteny, or an abundance of chromosomal rearrangements in otherwise closely related isolates. Other methods for detecting these processes include Dot Plots [18], or global alignment search tools such as Mummer [19] or Threaded Blockset Aligner (TBA) [20]. However, these methods are inherently genome rather than gene centric, requiring additional work to identify changes to gene content across species, or indeed distinguish erroneous ortholog or genome assembly from biological variation.

Synteny visualization has been implemented in a range of software suites and tools such as Sybil/Sybillite [21], which is both a command line and web tool to search and visualize several genomes based on clusters of orthologous genes. Another popular synteny visualization tool is Circos [22], which draws genomes as a circle, with arcs between regions of conservation or interactions. Owing to differences in requirements, data-input, and the type of visualization required – additional tools are still required for use in comparative genomes, while existing tools often require further development and maintenance for new features, and error corrections.

## Implementation

Here, I present a Perl based tool named Synteny Imager (Synima) to visualize chains of predicted orthologs between two or more genomes. Synima reads the orthology data contained in DAGchainer output files and generates and launches an Rscript visualising the locations and relationships between chromosomes and genes of each genome in PDF. Chromosomes and/or up to three separate gene categories can be optionally highlighted in a single run of Synima, either as specified on the command line from an initial run, or specified in a Synima config file. Synima is freely available from https://github.com/rhysf/Synima. Synima supersedes code that was successfully used in a range of projects [16, 23–25], where it facilitated the quantification and presentation of genome similarity and evolutionary changes between and within species. The tool has therefore been developed and tested on a range of datasets, including up to 12 genomes of 17.2–18.3 million bases long each, although this does not reflect an upper limit.

Included in the Synima package is a pipeline written for Linux or Macintosh OS for predicting and generating chains of orthologs between any number of genomes. Details of the methodology for each of these programs are available from their respective publications (BLAST [10], OrthoMCL [12] and DAGchainer [14]). Full details of the pipeline are also provided in the README accompanying the Synima application. Briefly, the Synima pipeline starts with a Repository specification file (Repo_spec) that specifies the genome FASTA, complementarity-determining region (cds) FASTA, peptide (pep) FASTA, and annotation GFF for each genome being compared. These files need to be (and are checked for being) uniformly formatted for each isolate or species (i.e. ID’s in FASTA corresponding to the same parent ID of a given feature in the GFF). The Repo_spec and accompanying files are used to generate a Repository Sequence Database, consisting of a summary of all the contained data, and are the input for the remaining steps. Next, an all vs all legacy BLAST wrapper script is run (optionally in parallel). The m8 formatted output from pairwise blasts are clustered using an OrthoMCL v1.4 wrapper script, that has the mcl application v10–201 dependency (https://micans.org/mcl/). Alternatively, (for very large datasets), the blast reports can be clustered by reciprocal best hits (RBH) with the Slclust application dependency (https://sourceforge.net/p/slclust/) that performs single-linkage clustering. Next, summaries of the OrthoMCL or RBH outputs are generated. A DAGchainer wrapper script is finally run on the. Cluster summary file, and Synima run on the DAGchainer output (.aligncoords and aligncoords.spans).

Synima (the ultimate step of the pipeline, or simply run on independently generated DAGchainer output) runs on the command line of Linux, Macintosh or terminal emulators in Windows, and requires only the Perl and R interpreters, and BioPerl installed. As input, Synima takes a genome FASTA file for each isolate of interest, and the predicted chains of orthologs in a tabulated delimited aligncoords and aligncoords.spans file, described in the README, which can be generated from tools such as DAGchainer [14]. Synima has a range of graphical parameters (size, chromosomal colours, gene colours, text etc.), and outputs a PDF overview of the determined synteny.

## Results

**Fig. 1:**
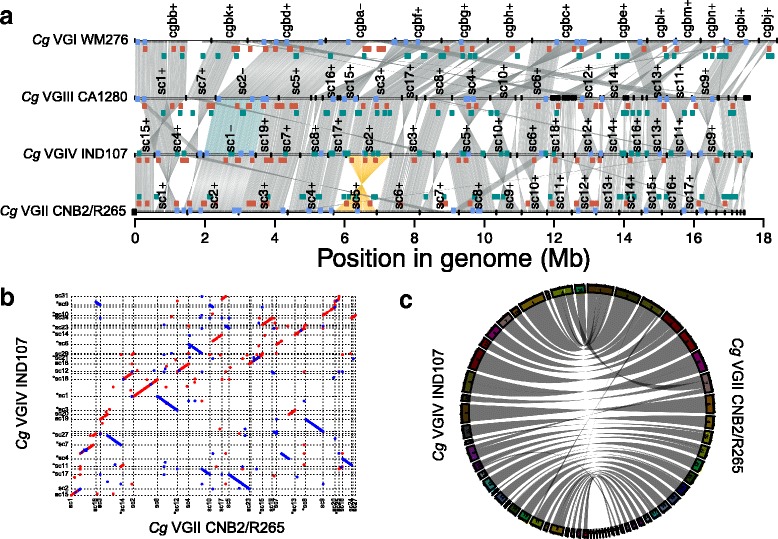
**a** Example output figure from Synima. Synteny is shown in the default R colour azure4 for four genomes representing each of the four lineages of the pathogenic fungus *Cryptococcus gattii* [16]. Isolate names are shown to the right of their genomes, which are represented by lines, with vertical lines indicating chromosomal/scaffold/contig borders, and their identifiers listed above (sc = supercontig, +/− = orientation). Supercontig (sc) 5 of the hypervirulent VGII CNB2/R265 isolate and sc1 of VGIV IND107 are highlighted in R colours ‘darkgoldenrod1’ and ‘cadetblue’, respectively. Genes involved in 1) ergosterol production, 2) capsule biosynthesis and 3) capsule attachment and cell wall remodeling [26] are shown as boxes in R colours ‘cornflowerblue’, ‘coral3’ and ‘darkcyan’, respectively. Sc’s and genes highlighted are for illustrative purposes only. The ordering and orientation of chromosomes are automatically calculated and applied by Synima, although manual changes to these can be made in the config file, e.g. re-orienting sc1 in IND107 (highlighted), CA1280 sc2 and WM276 cgba to avoid synteny overlap across the four genomes. **b** Mummer 3.22 alignment and Dotplot for *C. gattii* VGII CNB2/R265 vs *C. gattii* VGIV IND107. **c** Circos v0.66 figure of *C. gattii* VGII CNB2/R265 vs *C. gattii* VGIV IND107, ordered according to Synima’s pipeline

Figure 1a shows an example output figure from Synima. Here, the synteny (shown in default R colour ‘azure4’) of four genomes belonging to each of the four known lineages of the environmental and human pathogen *Cryptococcus gattii* are presented (data from [16]). Small black boxes above contig line show locations of all genes (And the lack of genes in a large region of WM276 cgbb is thereby revealed). For illustrative purposes, supercontig (sc) 5 of the hypervirulent VGII CNB2/R265 isolate and sc1 of VGIV IND107 are highlighted in R colours ‘darkgoldenrod1’ and ‘cadetblue’, respectively. The location of genes involved in 1. ergosterol production, 2. capsule biosynthesis and 3. capsule attachment and cell wall remodeling [26] are presented in R colours ‘cornflowerblue’, ‘coral3’ and ‘darkcyan’, respectively. As a comparison to Synima’s output, two alternative tools for visualizing synteny are shown in Fig. 1b and c: a Dotplot generated from a Mummer alignment [19] and a Circos figure respectively, both showing synteny between *C. gattii* CNB2/R265 and IND107. These alternative methods may be preferable for identifying chromosomal duplications within a genome for example, while Synima may be chosen for visualizing synteny between multiple genomes.

In addition to visualizing synteny, Synima includes a pipeline for the prediction of orthologs and preparing input aligncoords and aligncoords.spans files from a genome FASTA and annotation in GFF3 format for each isolate. The pipeline generates all vs all (pairwise) BLASTp hits with or without the option of parallel computing via the Platform Load Sharing Facility (LSF), Sun GridEngine (SGE) or Univa GridEngine (UGE), RBH or OrthoMCL clustering, and DAGChainer. Each program is included in the Synima repository, and was used for the generation of Fig. 1a. This pipeline therefore facilitates both the detection of orthologs, and the correctly formatted inputs for Synima.

## Conclusion

I present here a new tool for Synteny Imaging (Synima) from chains of predicted orthologs, including a pipeline for their prediction. Synima was used in several previous projects, although it has undergone large code refinements for reducing bugs, increased ability to run on a broad range of genome sizes (kilobases to megabases), FASTA ID formats, and a substantial increase in graphical parameters. For example, Synima identifies the clearest way to image the synteny with minimum overlap, which can nevertheless also be specified (or further refined) by editing the self-generated Config file.

Although several tools have been developed to visualize synteny from predicted orthologs i.e. [21, 22], the particular aesthetics of Synima’s output, its ease to which it can be incorporated into existing bioinformatics pipelines, and speed of use (circa minutes), should make Synima a valuable tool for researchers interested in synteny between two or more annotated genome assemblies, and highlighting genes of interest among them.
